# Effectiveness of the Comirnaty (BNT162b2, BioNTech/Pfizer) vaccine in preventing SARS-CoV-2 infection among healthcare workers, Treviso province, Veneto region, Italy, 27 December 2020 to 24 March 2021

**DOI:** 10.2807/1560-7917.ES.2021.26.17.2100420

**Published:** 2021-04-29

**Authors:** Massimo Fabiani, Mauro Ramigni, Valentina Gobbetto, Alberto Mateo-Urdiales, Patrizio Pezzotti, Cinzia Piovesan

**Affiliations:** 1 Department of Infectious Diseases, Istituto Superiore di Sanità, Rome, Italy; 2 Local Health Unit (ULSS 2 Marca Trevigiana), Treviso, Italy; 3 European Programme for Intervention Epidemiology Training (EPIET), European Centre for Disease Prevention and Control (ECDC), Stockholm, Sweden

**Keywords:** SARS-CoV-2 infection, COVID-19 disease, healthcare workers, vaccine effectiveness, Italy

## Abstract

Data on effectiveness of the BioNTech­/Pfizer COVID-19 vaccine in real-world settings are limited. In a study of 6,423 healthcare workers in Treviso Province, Italy, we estimated that, within the time intervals of 14–21 days from the first and at least 7 days from the second dose, vaccine effectiveness in preventing SARS-CoV-2 infection was 84% (95% confidence interval (CI): 40–96) and 95% (95% CI: 62–99), respectively. These results could support the ongoing vaccination campaigns by providing evidence for targeted communication.

By 24 March 2021, the coronavirus disease (COVID-19) pandemic has caused over 3.4 million cases and 105,000 deaths in Italy [1]. Although non-pharmaceutical interventions implemented in Italy were effective in reducing the impact of the first and second wave [2,3], there is urgency, now with the availability of approved vaccines, to accelerate the COVID-19 vaccination campaigns.

The first stage of the vaccination campaign in Italy started on 27 December 2020, which initially targeted healthcare workers (HCW) and residents in long-term care facilities. The Comirnaty, (BNT162b2, BioNTech/Pfizer, Mainz, Germany/New York, United States) vaccine was used because it was the only vaccine approved by the Italian Medicines Agency at that date [4]. Recommended administration was two doses 21 days apart.

Although efficacy of the Comirnaty vaccine has been proven in clinical trials [5], there is a need to evaluate its effectiveness in real-world settings. Based on surveillance data, this study aimed to estimate the effectiveness of the Comirnaty vaccine in preventing severe acute respiratory syndrome coronavirus 2 (SARS-CoV-2) infection in frontline HCW employed at the local health unit that serves the entire province of Treviso in the Veneto region (LHU-TV).

## Vaccination coverage and characteristics of healthcare workers included in the study

We conducted a retrospective cohort study of 9,878 HCW employed at the LHU-TV. From the local COVID-19 surveillance database, we retrieved information on demographic and professional characteristics, recorded dates of vaccine administration (all HCW were vaccinated with the Comirnaty vaccine) and the recorded date of SARS-CoV-2 infection, based on a positive antigenic test (SARS-CoV-2 Ag Test, LumiraDx, Alloa, United Kingdom (UK); sensitivity = 97.6% and specificity = 96.6% according to the manufacturer’s indications) confirmed by RT-PCR on the same day.

A total of 6,423 HCW were included in the analysis, after exclusion of 1,285 (13.0%) HCW infected with SARS-CoV-2 before the vaccination campaign, and 2,170 (22.0%) HCW working outside hospitals and district outpatient centres or who were support and administrative staff. The mean age of the included HCW was 47.1 years (standard deviation (SD): 10.8 years), most of them female (n = 4,986 (56.5%)) (Table 1). A total of 3,630 HCW were nurses (56.5%), 1,469 were medical doctors (22.9%), and 1,324 were social HCW (20.6%) (Table 1). All the included HCW were screened approximately every 8 days and at any other time if presenting symptoms consistent with COVID-19.

**Table 1:** Demographic and professional characteristics of healthcare workers by COVID-19 vaccination status, Treviso province, Italy, 27 December 2020–24 March 2021 (n = 6,423)

Characteristics	Unvaccinated		Vaccinated				Total	Adjusted RR^a^ of non-vaccination (95% CI)
			One dose		Two doses			
	n	%	n	%	n	%	n	
**Total**	**1,090**	**17.0**	**147**	**2.3**	**5,186**	**80.7**	**6,423**	NA
Sex								
Female	908	18.2	100	2.0	3,978	79.8	4,986	Ref.
Male	182	12.7	47	3.3	1,208	84.1	1,437	0.78 (0.67 to 0.92)
Age group								
< 30 years	82	14.9	17	3.1	451	82.0	550	0.96 (0.73 to 1.25)
30–39 years	261	22.5	33	2.8	866	74.7	1,160	1.57 (1.27 to 1.93)
40–49 years	261	16.7	35	2.2	1,267	81.1	1,563	1.07 (0.87 to 1.32)
50–54 years	233	15.3	40	2.6	1,252	82.1	1,525	0.89 (0.72 to 1.10)
55–59 years	146	15.8	14	1.5	763	82.7	923	0.91 (0.72 to 1.15)
≥ 60 years	107	15.2	8	1.1	587	83.6	702	Ref.
Professional category								
Nurse	638	17.6	72	2.0	2,920	80.4	3,630	Ref.
Medical doctor	166	11.3	40	2.7	1,263	86.0	1,469	0.64 (0.54 to 0.76)
Social HCW	286	21.6	35	2.6	1,003	75.8	1,324	1.28 (1.13 to 1.45)
Work context								
Hospital	791	16.3	112	2.3	3,937	81.3	4,840	Ref.
District outpatient centre	299	18.9	35	2.2	1,249	78.9	1,583	1.23 (1.09 to 1.39)

CI: confidence interval; COVID-19: coronavirus disease; HCW: healthcare workers; IQR: interquartile range; NA: not applicable; Ref.: reference; RR: relative risk.

^a^ Relative risks adjusted for all variables listed in the table estimated through a log-binomial regression model.

By 24 March 2021, 147 (2.3%) and 5,186 (80.7%) had received one and two doses of the Comirnaty vaccine, respectively, while 1,090 (17.0%) were still unvaccinated. The median time that elapsed between the administration of the two doses was 22 days (interquartile range (IQR): 21–24). The administration of the first vaccine dose occurred earlier among medical doctors (median date: 3 January 2021; IQR: 2–6 January 2021) compared with nurses (median date: 6 January 2021; IQR: 2–9 January 2021) and social HCW (median date: 6 January 2021; IQR: 3 January–9 February 2021).

The percentage of unvaccinated HCW was higher in women than in men (17.9% vs 13.5%), and in those aged 30–39 years (23.0%) compared with other age groups (Table 1). The highest percentage of complete vaccination with both doses was highest in medical doctors (85.7%) and HCW working in hospitals (82.1%).

## Cumulative probability of SARS-CoV-2 infection over time since the start of the vaccination campaign by vaccination status

We conducted a time-to-event analysis using the start of the vaccination campaign on 27 December 2020 as the index date. The length of the follow-up period was measured as the number of days that have elapsed from the index date to the estimated date of SARS-CoV-2 infection or until 24 March 2021, whichever came first. Vaccination was analysed as a time-dependent exposure, splitting individual records to account for the time duration as unvaccinated, vaccinated with only one dose, or vaccinated with both doses.

During the study period, a total of 242 (3.8%) HCW tested positive for SARS-CoV-2 infection. Of these, 171 (70.7%) developed symptoms.

**Figure 1:**
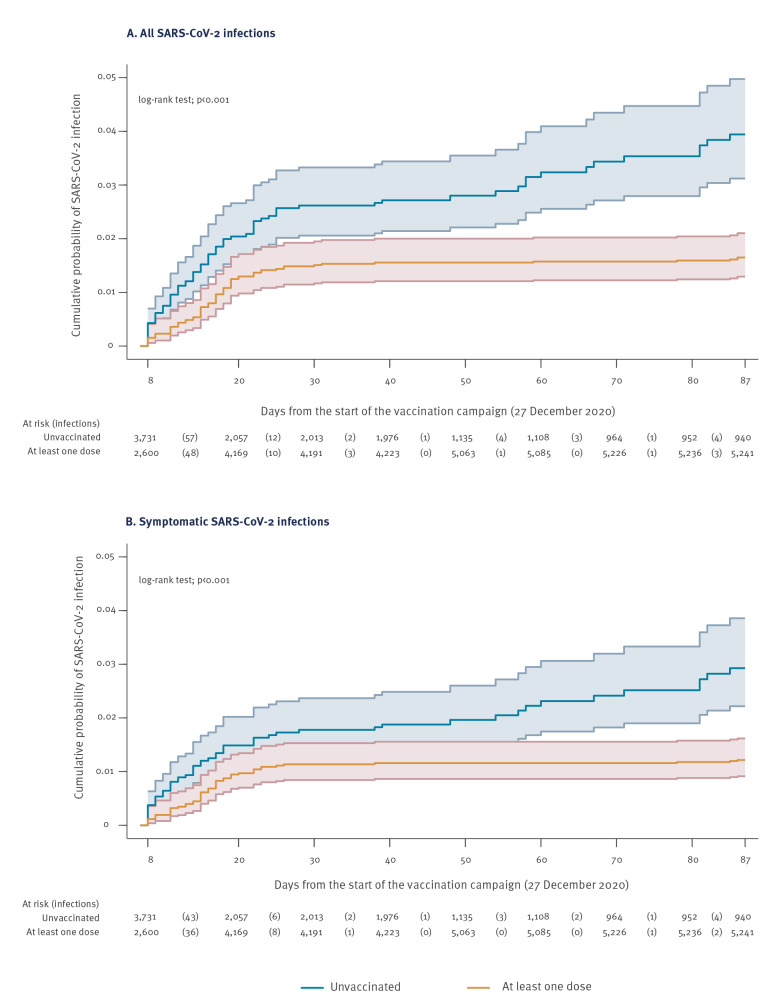
Kaplan–Meier failure curves of healthcare workers by COVID-19 vaccination status, Treviso province, Italy, 3 January–24 March 2021 (n = 6,331)^a^

COVID-19: coronavirus disease; HCW: healthcare workers; SARS-CoV-2: severe acute respiratory syndrome coronavirus 2.

^a^ The follow-up analysis was started on day 7 from the start of the vaccination campaign when the number of vaccinated HCW was sufficiently high to allow robust estimates [10].

Compared with unvaccinated HCW, the Kaplan–Meier failure curve showed a consistently reduced cumulative probability of SARS-CoV-2 infection in HCW who received at least one dose of the Comirnaty vaccine within 4 weeks from the start of the vaccination campaign (Figure 1A), and within ca 8 weeks when considering time to symptomatic infection as an outcome (Figure 1B). The Kaplan–Meier failure curves showing the cumulative probability of SARS-CoV-2 infection stratified by number of doses are presented in Supplementary Figure S1. 

Shaded bands delimit 95% confidence intervals. Data reported under the panels indicate the number of HCW at risk of infection at the start of each time interval and the number of infections (in brackets) that occurred within the time interval. Data are presented separately for unvaccinated HCW and those who received at least one dose of the vaccine.

## Effectiveness of the Comirnaty vaccine

We also conducted a time-to-event analysis using the number of days elapsed from vaccine administration to measure the length of follow-up. We estimated the effectiveness of one and two dose administration of the Comirnaty vaccine to prevent SARS-CoV-2 infection at different time intervals using a multivariable Cox proportional hazard model, including sex, age group, professional category, work context, and starting week of exposure as covariates. The adjusted hazard ratios (HR) were used to calculate vaccine effectiveness (VE) as ((1 − HR) × 100). 

In the time interval of 14–21 days after the administration of the first dose, VE in preventing all (both asymptomatic and symptomatic) and only symptomatic SARS-CoV-2 infections was estimated at 84% (95% CI: 40–96) and 83% (95% CI: 15–97), respectively (Table 2). In the time interval of at least 7 days after the administration of the second dose, VE increased to 95% (95% CI: 62–99) and 94% (95% CI: 51–99) in the two groups. The analysis showing the Kaplan-Meier failure curve by vaccination status according to time since vaccination or start of exposure for unvaccinated HCW is presented in Supplementary Figure S2.

**Table 2:** Comirnaty vaccine effectiveness in healthcare workers at various time intervals after COVID-19 vaccine administration, Treviso province, Italy, 27 December 2020–24 March 2021 (n = 6,423)**^a^**

Time of exposure	COVID-19 infections	PD	Incidence (per 1,000 PD)	Crude VE (%)	95% CI	Adjusted VE^b^ (%)	95% CI
**All cases^c^**							
**0–14 days**							
Unvaccinated	128	62,331	2.05	Ref.		Ref.	
Vaccinated - one dose	60	73,914	0.81	59.5	44.9 to 70.2	47.3	24.7 to 63.1
**14–21 days **							
Unvaccinated	15	14,496	1.03	Ref.		Ref.	
Vaccinated - one dose	6	36,600	0.16	84.1	59.1 to 93.8	84.1	39.7 to 95.8
**≥ 21 days **							
Unvaccinated	26	91,259	0.28	Ref.		Ref.	
Vaccinated - one dose	3	11,067	0.27	72.0	−27.8 to 93.9	85.4	−35.3 to 98.4
**0–7 days **							
Unvaccinated	11	14,186	0.78	Ref.		Ref.	
Vaccinated - two doses	0	35,596	0.00	NE		NE	
**≥ 7 days **							
Unvaccinated	15	77,073	0.19	Ref.		Ref.	
Vaccinated - two doses	4	216,098	0.02	90.2	68.7 to 96.9	95.1	62.4 to 99.4
**Symptomatic cases**							
**0–14 days **							
Unvaccinated	89	62,331	1.43	Ref.		Ref.	
Vaccinated - one dose	47	73,914	0.64	54.8	35.2 to 68.5	39.9	9.1 to 60.3
**14–21 days **							
Unvaccinated	8	14,496	0.55	Ref.		Ref.	
Vaccinated - one dose	4	36,600	0.11	80.1	34.0 to 94.0	83.3	14.8 to 96.7
**≥ 21 days **							
Unvaccinated	19	91,259	0.21	Ref.		Ref.	
Vaccinated - one dose	2	11,067	0.18	66.4	−153 to 95.5	65.9	−171 to 95.7
**0–7 days**							
Unvaccinated	6	14,186	0.42	Ref.		Ref.	
Vaccinated - two doses	0	35,596	0.00	NE		NE	
**≥ 7 days**							
Unvaccinated	13	77,073	0.17	Ref.		Ref.	
Vaccinated - two doses	2	216,098	0.01	94.1	73.3 to 98.7	93.7	50.8 to 99.2

CI: confidence interval; COVID-19: coronavirus disease; NE: not estimable (no cases among vaccinated healthcare workers); PD: person-days; Ref.: reference category; VE: vaccine effectiveness.

^a^ The analysis of VE for the second dose of the Comirnaty vaccine was performed beginning with the observation period on 17 January 2021 (i.e. the date of the first administration of the second dose).

^b^ VE adjusted for sex, age group, professional category, work context, and starting week of exposure.

^C^ Asymptomatic and symptomatic COVID-19 cases.

## Trend of COVID-19-associated hospital admissions in the study area, immunisation rate, and number of SARS-CoV-2 infections among healthcare workers

**Figure 2:**
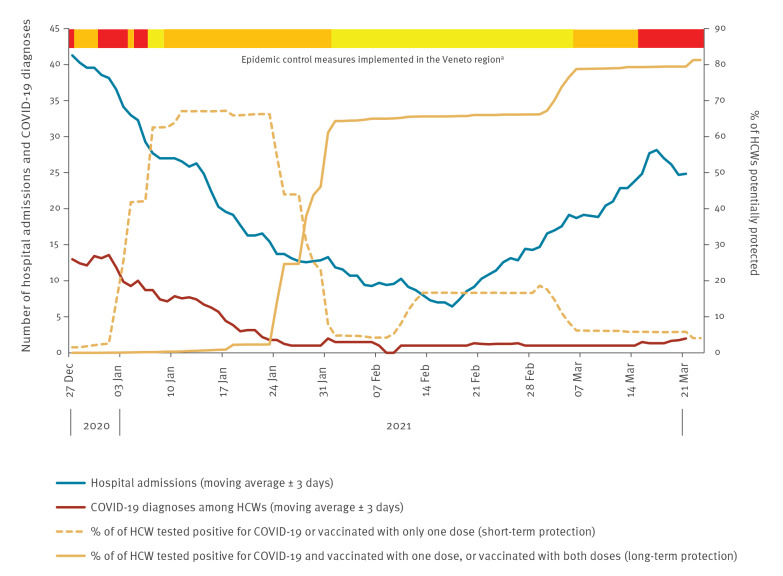
Trend of COVID-19-associated hospital admissions in the study area (n = 883,522 residents), and immunisation rate and number of SARS-CoV-2 infections among healthcare workers (n = 6,423), Treviso province, Italy, 27 December 2020–23 March 2021

COVID-19: coronavirus disease; HCW: healthcare workers; SARS-CoV-2: severe acute respiratory syndrome coronavirus 2.

^a^ The level of restrictions implemented in the Veneto region according to risk scenarios ranged from high (red band) to medium (orange band) and low (yellow band) [11].

Finally, we analysed the trend of COVID-19-associated hospital admissions in the study area, together with the trend of immunisation rate and of the number of SARS-CoV-2 infections among HCW included in the study. We found that from mid-February 2021, when the potential long-term immunisation rate among HCW was ca 70%, the number of newly diagnosed cases in this group remained stable despite a higher risk of exposure due to the rapid increase of COVID-19 hospital admissions (Figure 2).

## Ethical statement

The dissemination of COVID-19 surveillance data was authorised by the Italian Presidency of the Council of Ministers on 27 February 2020 (Ordinance no. 640).

## Discussion

Our analysis suggests that the Comirnaty vaccine had a high effectiveness in preventing SARS-CoV-2 infection in HCW during the time intervals after administration where protection may be expected [6]. Data on trends of COVID-19-associated hospital admissions in the study area, immunisation rate, and number of SARS-CoV-2 infections among HCW support this finding.

Moreover, also Italian national data suggest that the vaccination campaign in HCW was successful. A recent report has shown how, from mid-January 2021, COVID-19 incidence among HCW started to decrease rapidly, while it increased in the general population where vaccination coverage was still low [7].

To our knowledge, the few studies evaluating the Comirnaty vaccine effectiveness in preventing SARS-CoV-2 infection among HCW that included a control group were conducted in the UK and Israel [8,9]. We found that VE was 85% (95% CI: −35 to 98) after 21 days from the first dose administration compared with 72% (95% CI: 58–86) in the UK [8], although our estimate suffers from lack of precision because of the reduced time of follow-up in this interval (the large majority of subjects received the second dose of vaccine within few days from the scheduled date on day 21 from the first dose). When comparing VE 7 days after administration of the second dose, we found a higher VE of 95% (95% CI: 62–99) compared with 86% in the UK (95% CI: 76–97). Our estimates of VE in preventing symptomatic infections during the time intervals 1–14 days and 15–28 days from administration of the first dose were 40% (95% CI: 9–60) and 86% (95% CI: 33–97), respectively. This closely reflects the estimated VE of 47% (first dose, 95% CI: 17–66) and 85% (second dose, 95% CI: 71–92) estimated in Israel [9]. However, despite the general agreement, these comparisons should be interpreted with caution as they may be biased by several factors, such as differences in case definition and surveillance procedures.

Overall, 17% of the eligible HCW were not yet vaccinated almost 3 months after the start of the vaccination campaign, probably because of refusal. In accordance with findings from the UK, female individuals and HCW under 40 years of age had a lower tendency to be vaccinated, while medical doctors were the professional category showing the highest coverage [8].

This study has several limitations. It included only HCW and cannot be assumed as representative of the general population. We had no information about possible occurrence of adverse effects after vaccine administration, although there was no evidence of severe complications (no post-vaccination hospital admissions in vaccinated HCW). Unfortunately, the date of testing was not recorded in case of a negative result, and we were therefore unable to assess adherence to routine testing. However, the analyses evaluating VE in preventing all infections or only symptomatic infections did not show great differences. Given the probability that testing was performed in a timely manner in the event of symptoms, we feel confident that asymptomatic cases were also detected early. However, a residual differential bias could remain. For example, vaccinated people may have less rigorously adhered to testing, based on the belief they were protected, thus leading to an overestimate of VE. It is also possible that we missed mild or asymptomatic infections undetectable through the first-line antigenic test. It was not possible to accurately estimate VE in time intervals where the number of person-days of follow-up were much reduced (i.e. after 21 days from the administration of the first dose, before receiving the second dose). Finally, information about the SARS-CoV-2 variants linked to infections was not available and it was therefore not possible to evaluate VE according to this variable.

## Conclusions

In a real-world setting in northern Italy, during time intervals after vaccine administration where protection may be expected, we found a high effectiveness of the Comirnaty vaccine. This result could help to promote the ongoing vaccination campaign in the general population and among the still unvaccinated HCW by reinforcing communication based on evidence.

## Supplementary Data

747000 Supplement
