# Supplementary material for: Effectiveness of the Comirnaty (BNT162b2, BioNTech/Pfizer) vaccine in preventing SARS-CoV-2 infection among healthcare workers, Treviso province, Veneto region, Italy, 27 December 2020 to 24 March 2021
Source: Euro Surveill. 2021 Apr 29;26(17):2100420. doi: 10.2807/1560-7917.ES.2021.26.17.2100420 (PMC8086247; doi:10.2807/1560-7917.ES.2021.26.17.2100420)
Supplement: Supplement [file 21-00420_FABIANI_Supplement.pdf]

This supplementary material is hosted by Eurosurveillance as supporting information alongside the article "*Effectiveness of BNT162b2 (BioNTech-Pfizer) vaccine in preventing SARS-CoV-2 infection among health care workers in the Treviso province, Veneto region, Italy (27 December 2020 – 24 March 2021)*", on behalf of the authors, who remain responsible for the accuracy and appropriateness of the content. The same standards for ethics, copyright, attributions and permissions as for the article apply. Supplements are not edited by Eurosurveillance and the journal is not responsible for the maintenance of any links or email addresses provided therein.

Supplementary Figure S1. Kaplan-Meier failure curves of HCWs by vaccination status and dose according to calendar time, Treviso province, Italy, 3/24 January 2021 – 24 March 2021 (1 dose, n=6,331; 2 doses, n=6,204)[1].

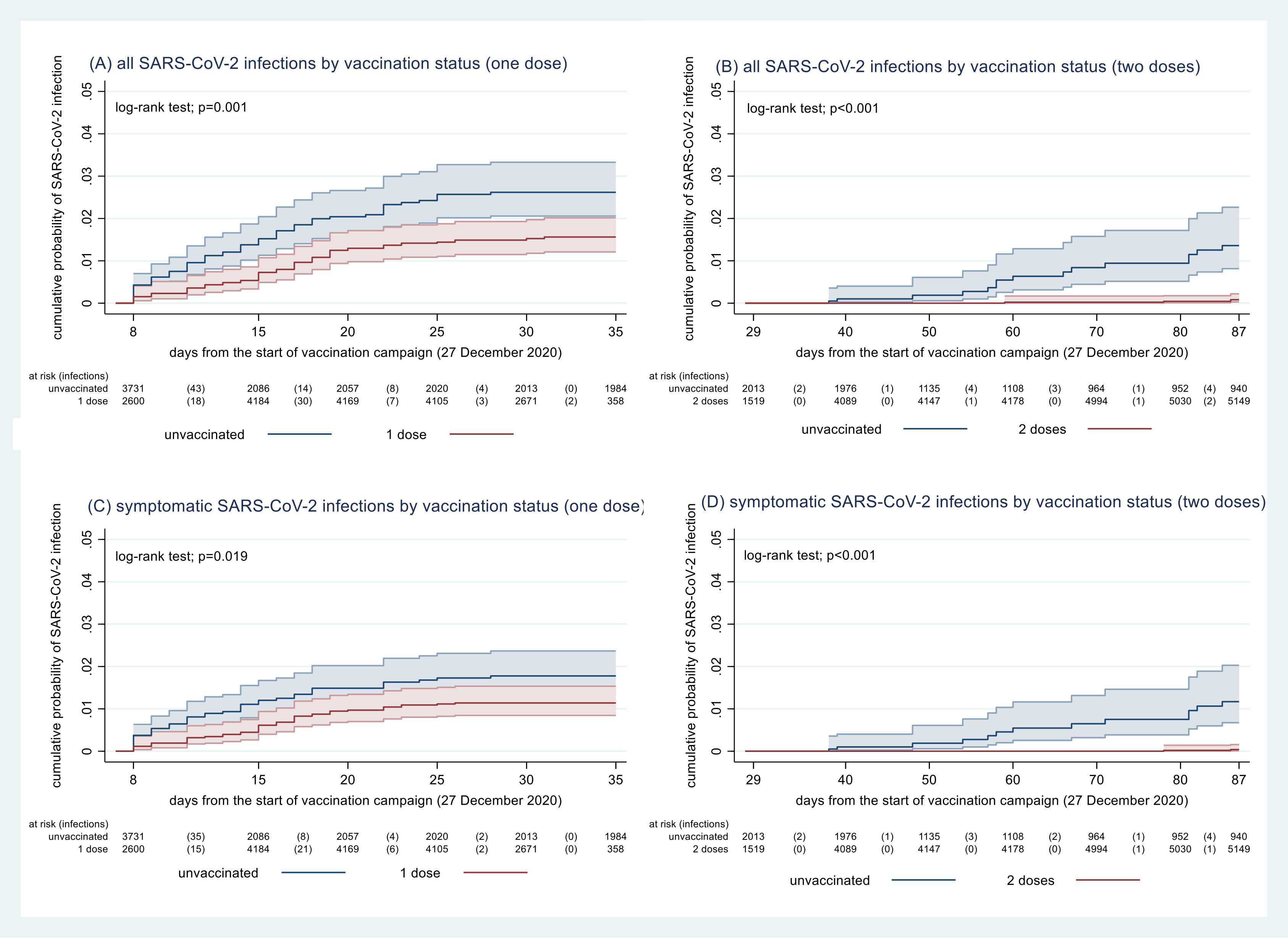

Shaded bands delimit 95% confidence intervals.

[1] The follow-up analysis was started on day 7/28 since the start of the vaccination campaign when the number of vaccinated HCWs with one and two doses was sufficiently high to allow robust estimates.

Supplementary Figure S2. Kaplan-Meier failure curves of HCWs by vaccination status and dose according to time since vaccination or start of exposure for unvaccinated HCWs, Treviso province, Italy, 27 December 2020/17 January 2021 – 24 March 2021 (1 dose, n=6,423; 2 doses, n=6,179[1]).

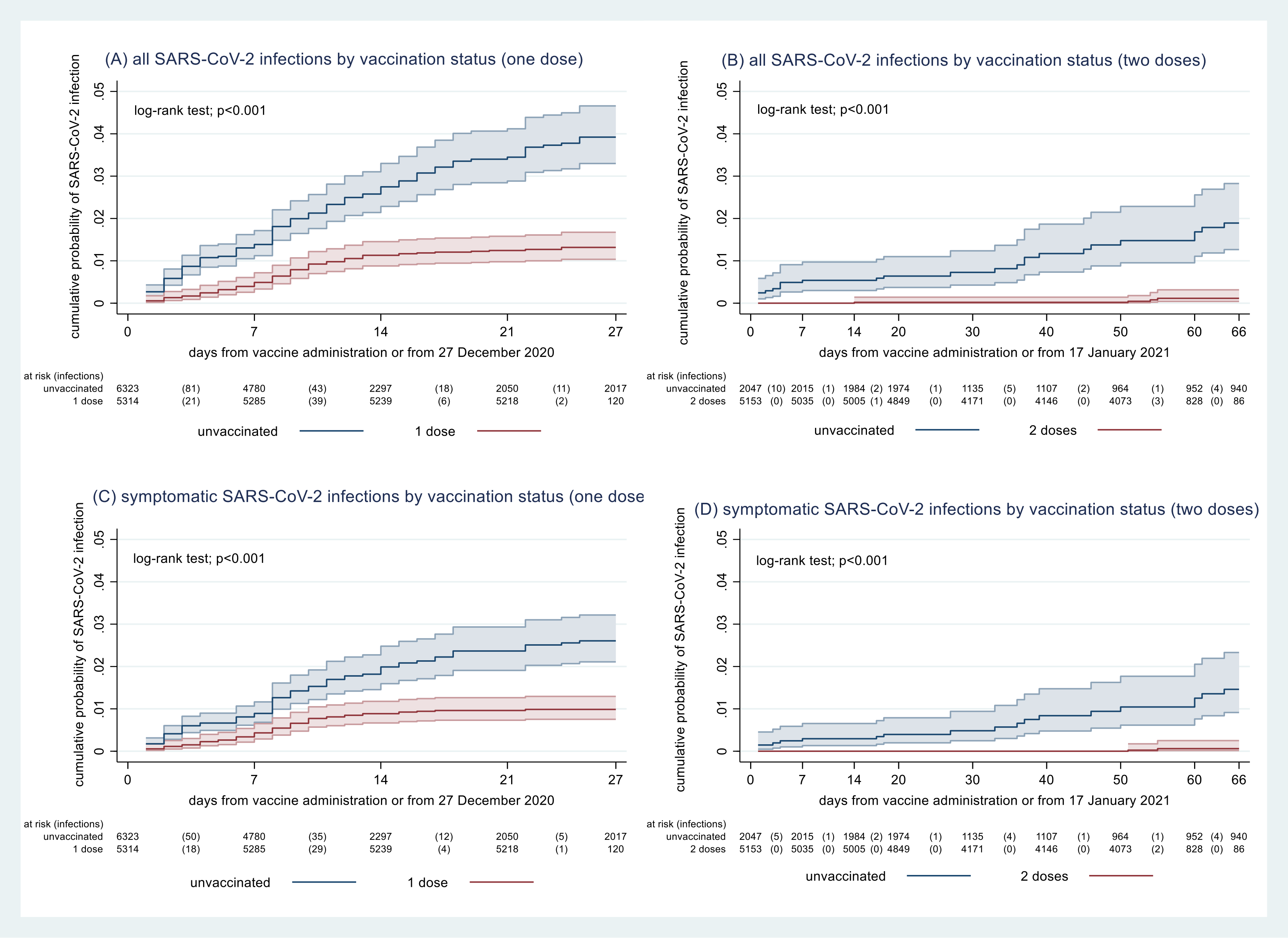

Shaded bands delimit 95% confidence intervals.

[1] The follow-up analysis for two doses vs. unvaccinated was started on 17 January 2021 (i.e., the date of first administration of the second dose).
